# Potent *In Vitro* Phosphodiesterase 1 Inhibition of Flavone Isolated from *Pistacia integerrima* Galls

**DOI:** 10.1155/2022/6116003

**Published:** 2022-01-17

**Authors:** Abdur Rauf, Sami Bawazeer, Jesús Herrera-Bravo, Muslim Raza, Humaira Naz, Somia Gul, Naveed Muhammad, Zainab M. Almarhoon, Yahia N. Mabkhot, Mohamed Fawzy Ramadan, William N. Setzer, Sevgi Durna Daştan, Shafi Mahmud, Javad Sharifi-Rad

**Affiliations:** ^1^Department of Chemistry, University of Swabi Anbar, 23430 Khyber Pakhtunkhwa, Pakistan; ^2^Department of Pharmacognosy, Faculty of Pharmacy, Umm Al-Qura University, Makkah, P.O. Box 42, Saudi Arabia; ^3^Departamento de Ciencias Básicas, Facultad de Ciencias, Universidad Santo Tomas, Chile; ^4^Center of Molecular Biology and Pharmacogenetics, Scientific and Technological Bioresource Nucleus, Universidad de La Frontera, Temuco 4811230, Chile; ^5^Departments of Chemistry, Bacha Khan University Charsadda, Khyber Pakhtunkhwa, Pakistan; ^6^Department of Zoology, Shaheed Benazir Bhutto Women University, Peshawar 2520, Pakistan; ^7^Faculty of Pharmacy, Jinnah University for Woman, Karachi, Pakistan; ^8^Department of Pharmacy, Abdul Wali Khan University, Mardan 23200, Pakistan; ^9^Department of Chemistry, College of Science, King Saud University, P.O. Box 2455, Riyadh 11451, Saudi Arabia; ^10^Department of Pharmaceutical Chemistry, College of Pharmacy, King Khalid University, Abha, Saudi Arabia; ^11^Deanship of Scientific Research, Umm Al-Qura University, Makkah, Saudi Arabia; ^12^Department of Chemistry, University of Alabama in Huntsville, Huntsville, AL 35899, USA; ^13^Aromatic Plant Research Center, 230 N 1200 E, Suite 100, Lehi, UT 84043, USA; ^14^Department of Biology, Faculty of Science, Sivas Cumhuriyet University, 58140 Sivas, Turkey; ^15^Beekeeping Development Application and Research Center, Sivas Cumhuriyet University, 58140 Sivas, Turkey; ^16^Genetic Engineering and Biotechnology, University of Rajshahi, Rajshahi, Bangladesh; ^17^Facultad de Medicina, Universidad del Azuay, Cuenca, Ecuador

## Abstract

To prospect an isozyme-specific, effective inhibitor against the physiologically-crucial enzyme phosphodiesterase 1 (PDE1), phytochemicals from *Pistacia integerrima* galls were screened. The chloroform fraction of gall extract was subjected to column chromatographic which led to the isolation of compound **1**, elucidated to be 5-hydroxy-7-methoxy-2-(4-methoxyphenyl)-4*H*-chromen-4-one (a flavone). *In vitro* and in silico PDE1 inhibitory activity of the compound **1** was investigated. EDTA, a known PDE1 inhibitor, was used as the reference. The flavone exhibited *in vitro* attenuation towards snake venom PDE1. IC_50_ response was superior to the standard chelator. An in silico molecular docking study was carried out using 3D structure of PDE1 to study the binding interactions of compound **1.** The docking study predicted that flavone had a lower binding affinity (-7.6 kcal/mol) and total energy (-95 kcal/mol) score compared to EDTA. The minimal energy associated with the ligand-protein complex implied that isolated compound 1 can serve as a therapeutic agent against PDE1 enzyme-provoked ailments like asthma, hypertension, schizophrenia, and erectile dysfunction.

## 1. Introduction

*Pistacia integerrima* J. L. Stewart ex Brandis, a member of the Anacardiaceae, is a species of pistachio tree, called zebrawood, native to Asia [1]. This medium-sized, deciduous tree grows at lower elevations (800-2400 m) of certain parts of the Himalayas [2]. The plant parts have been used to ameliorate fever, asthma, chronic bronchitis, dysentery, diarrhea, wounds, indigestion, jaundice, and worms [3]. The barks, leaves, and roots of *P. integerrima* have demonstrated efficacy against a range of maladies. The biological potency of *P. integerrima* galls developing on the leaves and petioles induced by an aphid *Pemphigus* sp. is interesting [4]. The hard and hollow galls have been verified to be a storehouse of functional compounds. The profusion of bioactive secondary metabolites in the galled tissue compared to normal plant tissues has been observed [5]. Ethyl acetate extract of *P. integerrima* plant exhibited high DPPH^∙^ radical-scavenging potential and demonstrated dose-dependent uric acid-lowering effect in hyperuricemic mice [6]. Extract of *P. integerrima* was active against many Gram-positive and two Gram-negative pathogens [7]. An active constituent of *P. integerrima* gall was identified as ethyl gallate. This ethyl ester of gallic acid lowered lipopolysaccharide-induced cell adhesion molecule (CAM) expression by blocking activator protein 1 (AP-1) transcription factor [8]. A triterpene, pistagremic acid (PA) isolated from the galls exhibited strong leishmanicidal activity, wherein the lethality against *Leishmania* major promastigotes was less than amphotericin B [9]. PA attenuated the activity of both yeast and rat intestinal *α*-glucosidases [10]. Intraperitoneal administration of PA (10 mg/kg) was shown to exhibit antinociceptive effect in the acetic acid-induced writhing test. In the tail immersion test, pretreatment with the triterpene (10 mg/kg), pain reduction was 59.4%. Additionally, it alleviated carrageenan-induced edema. Antipyretic effect was observed for 10 mg/kg administration to mice. A molecular docking study revealed that PA fits well in the active site of COX-2 enzyme due to hydrogen-bonding and hydrophobic moiety interactions [10, 11].

An *in vitro* study exhibited the antiradical potential of solvent fractions of *P. integerrima* leaf ethanol extract [12]. Essential oil of the galls in the treatment of bronchial asthma is possibly related to its ability to inhibit L-subtype Cav channel, mast cell stabilization, antioxidant, angiogenesis, and the inhibition of 5-lipoxygenase [3]. Apart from the rich polyphenolic profile, acylated oligosaccharides (integrisides A and B) were isolated from the butanol fraction of methanolic extract of *P. integerrima* [13]. Investigations unveiled that the ethyl acetate and chloroform fractions of *P. integerrima* confer strong cytotoxicity towards human breast cancer (MCF-7) cell line.

The current study deals with the isolation and purification of an efficacious phosphodiesterase-1 (PDE1) inhibitor from *P. integerrima* followed by docking studies.

## 2. Experimental

### 2.1. Sample Collection

*P. integerrima* galls were collected from Sultan Muhammad Razagram garden (district Dir, K.P.K., Pakistan). The botanical sample was verified by Prof. Dr. A. Rashid (Botany Department, University of Peshawar, Pakistan). A voucher specimen (Bot 20037, PUP) was deposited in the department.

### 2.2. Extraction and Isolation

**Figure 1:**
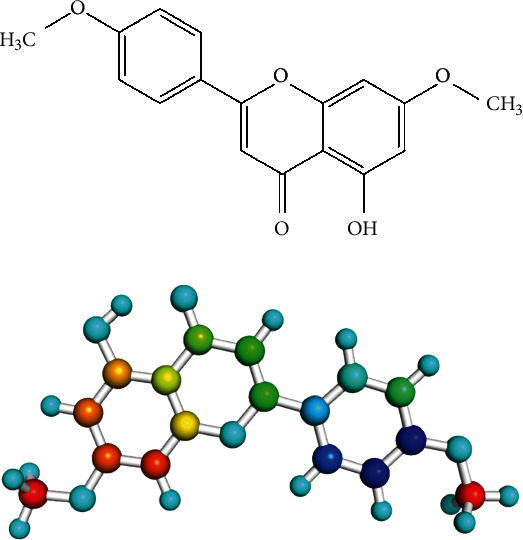
Structure of compound 1 [5-hydroxy-7-methoxy-2-(4-methoxyphenyl)-4*H*-chromen-4-one]; (a) 2D and (b) 3D ball and stick model.

Shade-dried and crushed galls of *P. integerrima* were extracted with methanol. The extract was suspended in water and sequentially fractionated with *n*-hexane, chloroform, and ethyl acetate. The green residue from the chloroform fraction was subjected to column chromatography using silica gel. The elution of column was done using a mixture of methanol : chloroform (1 : 9, *v*/*v*). The fractions were combined according to their TLC profile into 10 different subfractions (PS-1 to PS-10). Fraction PS-10 was further subjected to repeated column chromatography and TLC, which furnished the compound 5-hydroxy-7-methoxy-2-(4-methoxyphenyl)-4*H*-chromen-4-one (compound **1**). The structure of compound 1 (Figure 1) was confirmed by comparing its physical and spectral data with the previously reported compound [14].

### 2.3. Phosphodiesterase 1 Inhibition Assay

The activity of compound **1** against snake venom PDE1 (Sigma P-3134 manufactured from pit viper venom, EC 3.1.4.1) was assayed [15]. Tris-HCl buffer 33 mM (pH 8.8) and 30 mM magnesium acetate (cofactor) were added to 0.000742 U of PDE1 as a final concentration using a 96-well flat bottom plate and 0.33 mM bis-(*p*-nitrophenyl) phosphate (Sigma N-3002) as a substrate. Ethylenediamine tetra acetic acid (Merck), a chelating agent, was used as positive control. After 30 min of incubation, the enzyme activity was monitored at 37°C on a microtitre plate reader spectrophotometer (Molecular Devices, USA) by measuring the release of *p*-nitrophenol (a chromogenic product) from *p*-nitrophenyl phosphate (a nonproteinaceous, nonspecific substrate) at 410 nm. All the reactions were performed in triplicate, and the initial rates were measured as the rates of changes in the optical density (OD)/min and used in subsequent calculations [16].

(1) $$\begin{array}{c} \%\text{Inhibition}=100-\left(\frac{{\text{OD}}_{\text{testwell}}}{{\text{OD}}_{\text{control}}}\right)\times 100. \end{array}$$

### 2.4. Homology Modeling

The snake venom PDE1 sequence was retrieved from Uniprot database (accession number of J3SEZ3) [17]. The template selection was carried out by BLAST against the Protein Data Bank (PDB) database [14, 15]. The crystal structure of *Mus musculus* (four-letter code: 4GTW) was obtained as the best hit according to its sequence identity [18]. The alignment of template and target sequence was carried out through BioEdit software [19]. The 3D model of PDE1 was generated through MODELLER 9.12 [20]. The predicted models were refined using energy optimization through Swiss PDB viewer v4.1.0 software [21]. The 3D structures were evaluated by ProSA [22] and Procheck online servers (Figure S1 a, b, and c) [23]. The best predicted model was selected for molecular docking studies.

### 2.5. Docking Studies

Docking is the in silico prediction of the most favorable conformation of proteins in a complex by calculating the energy contained in the system, with the most accurate structure having the lowest energy [18]. The docking study for predicted model was carried out using two docking software, iGEMDOCK v 2.1 [19] and AutoDock Vina [20]. The docking procedure of both software was calibrated by already docked ligand in the receptor file. The 3D structure of compound 1 and EDTA was prepared by using Chem Draw [21]. The ligand was prepared through adjusting the chemical correctness (protonation) and stereochemical and ionization variation using computational tools. All the default parameters of iGEMDOCK software were used for the docking of compound 1 and EDTA against PDE1. The receptor file was cleaned from solvent molecules followed by hydrogen addition and Gasteiger partial charge calculation. The PDE1 3D model and ligands were uploaded in the PyRx tool [22]. The receptor and compound files were converted into PDBQT format (that holds information for the atomic coordinates, partial charges and solvation parameters for all the atoms in the molecule). The grid center of grid box was placed on the active site of the PDE1 receptor. The grid box of center *x* = 30.28, *y* = 32.70, and *z* = 23.05 and size of *x* = 43.85 Å, *y* = 45.40 Å, and *z* = 49.29 Å with an exhaustiveness global search algorithm was set up to 8. The docked poses of predicted model PDE1 with compound 1 were analyzed by Discovery studio visualizer version 4.0 [24], PyMOL version 1.7.2 [25], and LIGPLOT+ version v.1.4.5 software [23, 26].

## 3. Results

### 3.1. Results of PDE-1 Inhibition

The current study revealed the PDE1 inhibition potency of 5-hydroxy-7-methoxy-2-(4-methoxyphenyl)-4*H*-chromen-4-one (5-hydroxy-4′,7-dimethoxy-flavone) isolated from *P. integerrima* galls. As shown in Table 1, the flavone elicited 93.9% inhibition at 0.2 mM concentration with outstanding potency (IC_50_ 13.55 *μ*M). EDTA exhibited 80.1% inhibition with IC_50_ 276.1 *μ*M.

### 3.2. Docking Studies

**Figure 2:**
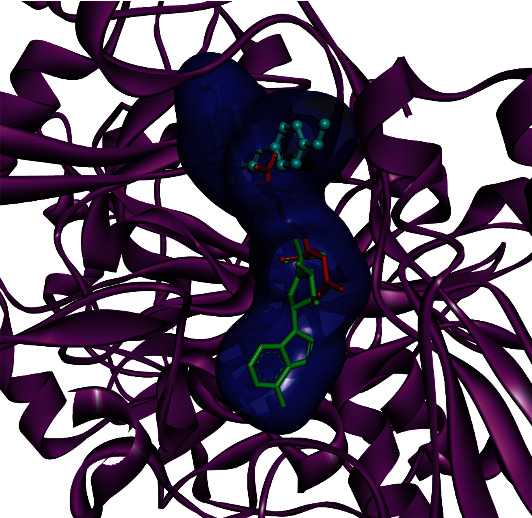
PDE1 predicted docked poses of compound 1 with ball and stick (cyan color), EDTA with stick (red color), and cocrystallized ligand with stick (green color).

**Figure 3:**
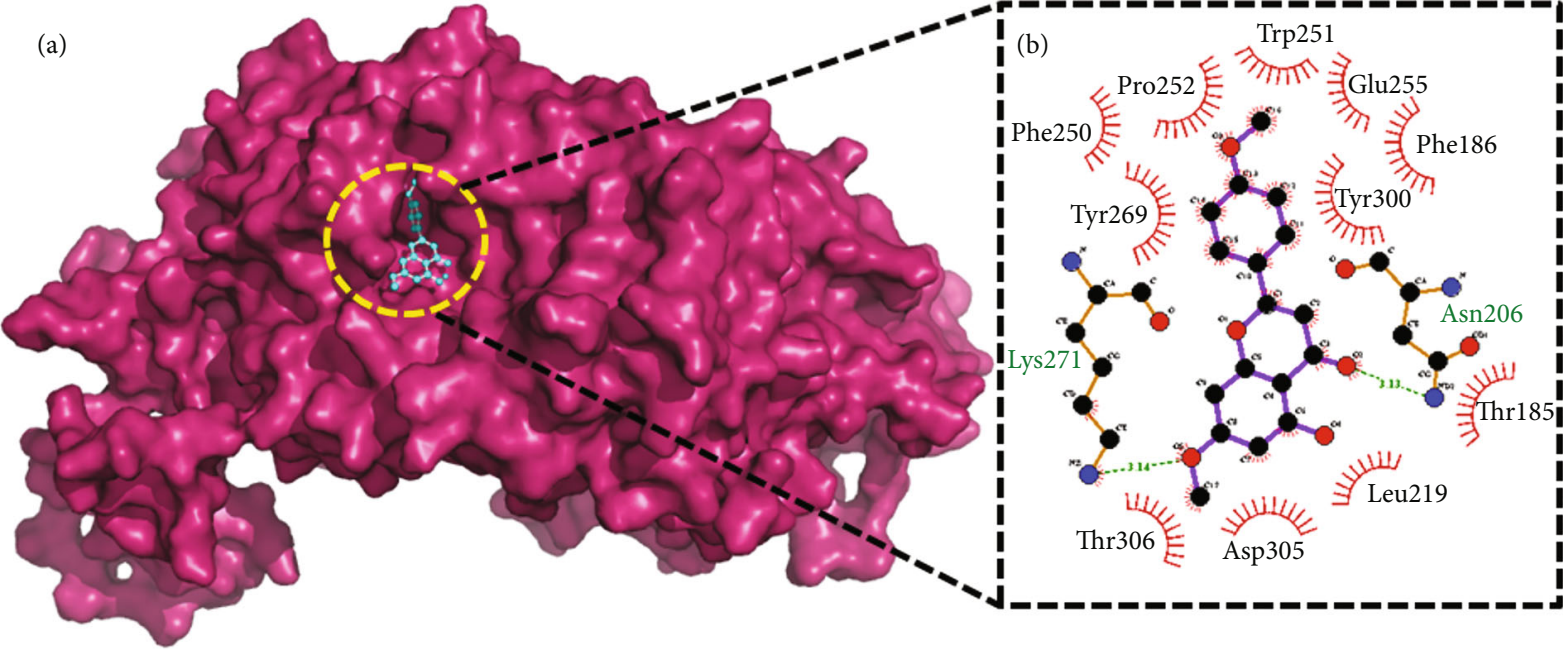
(a) refers to the predicted dock pose of compound (1) with PDE1 while (b) displays the detailed schematic presentation of docked compound 1 against venom PDE1 with labelling of hydrogen bonding and hydrophobic interaction.

The analysis of docking simulation was carried out based upon the hydrogen bond and hydrophobic interactions. The interaction analysis indicated that compound 1 has good docking results compared to EDTA against PDE1. The docking result of compound 1 and EDTA are presented in Table 2. The predicted docking poses and superimposition of the compound 1 along with EDTA are shown in Figure 2. The interaction analysis of compound 1 (Figure 3) indicated that it forms two hydrogen bond interactions with Asn206 and Lys271 with a distance of 3.13 Å and 3.14 Å, respectively. Compound 1 also formed eleven hydrophobic contacts with residues Thr185, Phe186, Leu219, Phe250, Trp251, Pro252, Glu255, Tyr269, Tyr300, Asp305, and Thr306. AutoDock Vina binding energy score for compound 1 was -7.6 kcal/mol, while it was -6.5 kcal/mol for EDTA (Table 2). The iGEMDOCK total energy score for compound 1 was -95 kcal/mol, while it was -89 kcal/mol for EDTA (Table 2). The lower energy associated with compound 1 indicates its superior interacting capacity PDE1 receptor, compared to EDTA. This finding appears interesting in this regard as the docking result proved the lowest energy associated with the ligand-protein binding. The isolated flavone merits *in vivo* assessment.

## 4. Discussion

Phosphodiesterases (PDEs) are nonspecific exonucleases, capable of hydrolyzing DNA, RNA, ATP, ADP, and NAD [27]. They catalyze the hydrolysis of phosphodiester bonds in cAMP and cGMP [28]. There are 11 different families of PDEs with specific substrate kinetic properties, mode of regulation, intracellular localization, and tissue expression patterns [29, 30]. They mediate diverse pathways such as the metabolism of extracellular nucleotides (nuclease activity), hydrolysis of nucleoside 5′-triphosphates (phosphatase activity), and regulation of nucleotide-based intercellular signaling. This enzyme in snake venom (vipers) perturbs homeostasis of the victim and aggregates plasma platelets causing blood coagulation. Hyperactivity of PDEs has been verified to drive arthritis, hypertension, and cardiovascular diseases [31]. Antivenom serum and metal chelator EDTA are known to inactivate PDEs [32]. Novel PDE inhibitors are sought after as a treatment for a range of inflammatory diseases. Natural phytochemical inhibitors have been reported to offer pharmacological properties such as smooth muscle relaxant, bronchodilator, vasodilator, antidepressant, antithrombotic, anti-inflammation, and cognitive promoter [30]. Methylxanthine was the first natural inhibitor of cyclic nucleotide PDEs. All PDE inhibitors contain one or more rings that mimic the purine in the cyclic nucleotide substrate and directly compete with it for access to the catalytic site [33]. Nobiletin, a polymethoxylated flavonoid extracted from citrus rind inhibited PDEs, elevating AMP level, and dopamine release. It assuaged memory malfunctioning in induced-Parkinson model mice [34]. *Forsythia suspensa* seed-derived lignin inhibited PDE4 in inflammatory and immune cells [35]. The mechanism was elucidated as the denaturation of PDE enzymes that influences cyclic adenosine monophosphate cAMP. An insightful review sheds light on the potency of PDE inhibitors to restore normalcy in dopamine signaling and thus treat motor, cognitive, and psychiatric disorders [29]. The provocative role of PDEs in airway smooth muscle inflammation and latest achievements in asthma and chronic obstructive pulmonary disease (COPD) management by targeting the enzyme was reviewed [36]. Drugs inhibiting PDE activity have a therapeutic action on the heart, lung, and vasculature as well as on platelet function and inflammatory mechanisms. These tissues express more multiple isoenzymes of PDEs. Thus, the drugs target more than one isoenzyme, which has undesirable consequences. For example, cilomilast ameliorates pulmonary functions but sets off gastrointestinal disturbances, emesis, and nausea [37]. Similarly, theophylline is used to treat respiratory problems, but when prescribed at higher concentrations, it causes cardiac arrhythmias and seizures [38]. It makes the quest for isoenzyme-selective inhibitors relevant. *In silico* docking studies were conducted for comparative modeling of PDE1 from snake venom, selected as the best predictive model. The molecular docking study of PDE1 was carried out through two docking software (iGEMDOCK and AutoDock Vina). The aim of this study was to validate the *in vitro* studies and identify the inhibitory specificity of compound 1 and efficacy in comparison to EDTA. Weak intermolecular interactions such as hydrogen bonding and hydrophobic interactions are crucial in stabilizing energetically favored ligands in protein structures. Therefore, quantification of the interaction energy between the target 3D structures and the ligand molecules is important to determine the affinity [17].

## Figures and Tables

**Table 1:** Enzymes inhibitory activities of compound 1 and EDTA.

Compound	PDE1 inhibition (%) (0.2 mM)	IC_50_ ± S.E.M. (*μ*M)
1	93.91	13.55 ± 0.04
EDTA	80.10	276.1 ± 2.65

**Table 2:** The docking score and interaction profile of compound 1 and EDTA.

Compound	AutoDock Vina (kcal/mol)	GEM DOCK (kcal/mol)		Molecular interactions for compound 1	
				Hydrophobic	Hydrogen bonding
	B. Affinity	Total energy	VDW		
1	-7.6	-95	-82	Thr185, Phe186, Leu219, Phe250, Trp251, Pro252, Glu255, Tyr269, Tyr300, Asp305, and Thr306	Asn206 (3.13 Å) Lys271 (3.14 Å)
EDTA	-6.5	-89	-60		

## Data Availability

The data used to support the findings of this study are available from the corresponding author upon request.

## Supplementary Materials

Supplementary Materials Figure S1: (a) and (b) refer to the ProSA result, and (c) represent the ProCheck result for best model evaluation and selection.
