# Supplementary material for: Potent In Vitro Phosphodiesterase 1 Inhibition of Flavone Isolated from Pistacia integerrima Galls
Source: Biomed Res Int. 2022 Jan 17;2022:6116003. doi: 10.1155/2022/6116003 (PMC8786535; doi:10.1155/2022/6116003)
Supplement: Supplementary Materials — Figure S1: (a) and (b) refer to the ProSA result, and (c) represent the ProCheck result for best model evaluation and selection. [file 6116003.f1.docx]

**SUPPLEMENTARY MATERIAL**

**Potent *in vitro* Phosphodiesterase 1 Inhibition of Flavone Isolated from *Pistacia integerrima* Galls**

Abdur Rauf ^1*^, Sami Bawazeer^2^, Jesús Herrera-Bravo^3,4^, Muslim Raza^5^, Himaira Naz^6^, Somia Gul^7^, Naveed Muhammad^8^, Zainab M. Almarhoon^9^, Yahia N. Mabkhot^10^, Mohamed Fawzy Ramadan^11^, William N. Setzer^12^, Sevgi Durna Daştan^13,14^, Shafi Mahmud^15^, Javad Sharifi-Rad^16*^

^1^Department of Chemistry, University of Swabi Anbar-23430, Khyber Pakhtunkhwa, Pakistan

^2^Department of Pharmacognosy, Faculty of Pharmacy, Umm Al-Qura University, Makkah, P.O. Box 42, Saudi Arabia

^3^Departamento de Ciencias Básicas, Facultad de Ciencias, Universidad Santo Tomas, Chile

^4^Center of Molecular Biology and Pharmacogenetics, Scientific and Technological Bioresource Nucleus, Universidad de La Frontera, Temuco, 4811230, Chile

^5^Departments of Chemistry, Bacha Khan University Charsada, Khyber Pakhtunkhwa, Pakistan

^6^Department of Zoology, Shaheed Benazir BhuttoWoman University Peshawar, 2520, Pakistan;

^7^Faculty of Pharmacy Jinnah University for Woman, Karachi, Pakistan

^8^Department of Pharmacy, Abdul Wali Khan University, Mardan 23200, Pakistan

^9^Department of Chemistry, College of Science, King Saud University, P.O. Box 2455, Riyadh 11451, Saudi Arabia

^10^Department of Pharmaceutical Chemistry, College of Pharmacy, King Khalid University, Abha, Saudi Arabia

^11^Biochemistry Department, Faculty of Agriculture, Zagazig University, Zagazig 44519, Zagazig, Egypt

^12^Department of Chemistry, University of Alabama in Huntsville, Huntsville, AL 35899, USA ^10^Aromatic Plant Research Center, 230 N 1200 E, Suite 100, Lehi, UT 84043, USA

^13^Department of Biology, Faculty of Science, Sivas Cumhuriyet University, 58140, Sivas, Turkey

^14^Beekeeping Development Application and Research Center, Sivas Cumhuriyet University, 58140, Sivas, Turkey

^15^Genetic Engineering and Biotechnology, University of Rajshahi, Rajshahi, Bangladesh

^16^Phytochemistry Research Center, Shahid Beheshti University of Medical Sciences, Tehran, Iran

*Corresponding authors: [mashaljcs@yahoo.com](mailto:mashaljcs@yahoo.com) (A.R.);javad.sharifirad@gmail.com (J.S.-R.)


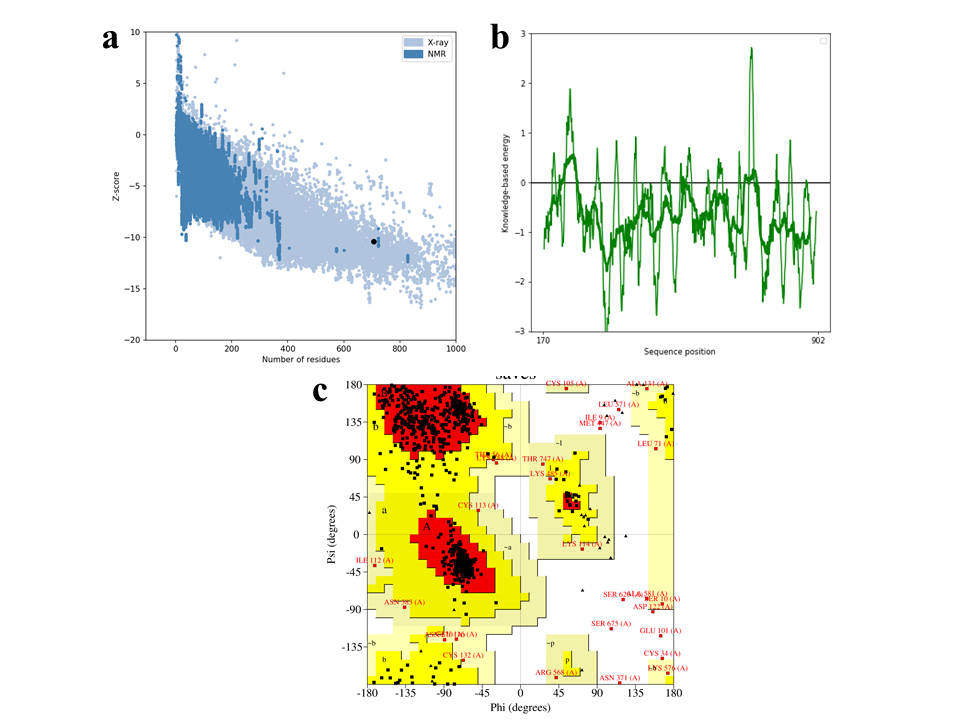


**Figure S1:** (a) and (b) refer to the ProSA result and (c) represent the ProCheck result for best model evaluation and selection.
